# Premyopia Management With Ophthalmic Referral Slows Myopic Shift After School Entry: A Population‐Based Longitudinal Study in Taiwan

**DOI:** 10.1111/ceo.14595

**Published:** 2025-08-09

**Authors:** Yu‐Chieh Yang, Hsin‐Yu Yang, Chiao‐Yu Wang, Shao‐You Fang, Chia‐Wei Lee, Pei‐Wei Huang, Mong‐Ping Shyong, Yen‐Lin Chen, Nai‐Wei Hsu, Der‐Chong Tsai

**Affiliations:** ^1^ Department of Ophthalmology National Yang Ming Chiao Tung University Hospital Yilan Taiwan; ^2^ Department of Ophthalmology Taipei Veterans General Hospital Taipei Taiwan; ^3^ Faculty of Medicine National Yang Ming Chiao Tung University School of Medicine Taipei Taiwan; ^4^ Department of Ophthalmology, Yuanshan and Suao Branch Taipei Veterans General Hospital Yilan Taiwan; ^5^ Institute of Clinical Medicine National Yang Ming Chiao Tung University Taipei Taiwan; ^6^ University of California Davis Center for Healthcare Policy and Research Sacramento California USA; ^7^ Department of Ophthalmology, Fu Jen Catholic University Hospital Fu Jen Catholic University New Taipei City Taiwan; ^8^ School of Medicine, College of Medicine Fu Jen Catholic University New Taipei City Taiwan; ^9^ Department of Ophthalmology, Lotung Poh‐Ai Hospital Lo‐Hsu Medical Foundation Incorporation Yilan Taiwan; ^10^ Public Health Bureau Yilan Taiwan; ^11^ Division of Cardiology, Department of Internal Medicine National Yang Ming Chiao Tung University Hospital Yilan Taiwan; ^12^ Community Medicine Research Center & Institute of Public Health National Yang Ming Chiao Tung University Taipei Taiwan

**Keywords:** myopia progression, ophthalmic referral, premyopia, preschooler

## Abstract

**Background:**

This study aimed to evaluate the impact of early ophthalmic referral for premyopic preschoolers on myopic spherical equivalent (SE) shift after school entry; and to identify risk factors for nonadherence to follow‐up care.

**Methods:**

A population‐based longitudinal study followed 742 premyopic preschoolers (−0.5 D < SE ≤ +0.75 D) from a countywide screening in Yilan, Taiwan (2021–2022), into first or second grade in elementary school (2023). Cycloplegic autorefraction and caregiver questionnaires were collected at baseline and follow‐up. Children screened in 2022 comprised the intervention cohort, while those screened in 2021 formed the comparison cohort. Multiple regression analyses assessed factors associated with myopic SE shift and nonadherence to follow‐up care.

**Results:**

Amongst 742 premyopic children (mean [SD] age at follow‐up, 7.2 [0.4] years; 57.8% boys), the overall SE progression was −0.19 (0.39) D/year and the incidence of myopia was 14.8 per 100 person‐years. The intervention cohort had a slower myopic SE shift than the comparison cohort (−0.15 vs. −0.22 D/year; *p* = 0.009). Referral at baseline was independently associated with slower SE progression (*β* = 0.070; *p* = 0.03). Amongst those referred in the intervention cohort, 63.2% adhered to follow‐up care. Nonadherence was associated with greater baseline hyperopia (adjusted OR, 3.77; 95% CI, 1.69–8.41) and rural residency (adjusted OR, 2.01; 95% CI, 1.23–3.30).

**Conclusions:**

Early ophthalmic referral reduced myopic progression, but follow‐up adherence was suboptimal, especially in children with greater hyperopia or living in rural areas.

## 1 Introduction

Juvenile‐onset myopia may be associated with a rapid progression to high myopia in adulthood [1]. High myopia increases the risk of developing pathologic changes—such as myopic maculopathy, retinal detachment, cataracts and glaucoma—which can impose a substantial burden on the healthcare system and health insurance infrastructure [2]. From a preventive medicine perspective, adopting strategies to delay the onset of myopia and slow its progression is crucial, particularly for young children at high risk of developing myopia.

Premyopia, defined as a cycloplegic refractive state in children that is more hyperopic than −0.5 D and not more hyperopic than +0.75 D (−0.5 D < SE ≤ +0.75 D), was proposed as a recognised refractive group by the International Myopia Institute in 2019 as part of efforts to prevent myopia [3, 4]. Premyopia has been identified as a significant risk factor for the onset of myopia amongst schoolchildren [5]. Our previous study in Yilan, Taiwan, revealed that premyopia is prevalent amongst preschool children and is associated with environmental factors such as increased screen time and reduced outdoor activities [6]. This finding suggests that the factors contributing to myopia development may already be active before premyopic children reach school age.

Spending more time outdoors has been demonstrated as a protective factor against the onset of myopia [7, 8]. Since the implementation of a government policy promoting outdoor activities (Tian‐Tian 120 programme) in 2010, the previously rising trend of low visual acuity (VA) prevalence has been reversed amongst schoolchildren in Taiwan [9]. Nevertheless, childhood myopia prevalence has been reported to rise significantly around elementary school entry, increasing from 9.0% in kindergarteners to 19.8% in first graders and 38.7% in second graders [10]. In 2022, to enhance the prevention strategy for early‐onset myopia, the Public Health Bureau of Yilan, Taiwan, began referring preschoolers identified with premyopia during the Yilan Myopia Prevention and Vision Improvement Program (YMVIP), a preschool screening programme initiated in 2014 [11], for regular ophthalmic care.

Early identification and eye‐care referrals may help premyopic preschoolers at high risk for myopia onset regularly monitor spherical equivalent (SE) refractive error changes and receive timely myopia intervention if needed. Although few clinical trials have focused specifically on delaying the onset or progression of myopia in premyopic children, the Low‐Concentration Atropine for Myopia Progression (LAMP2) study in Hong Kong confirmed that the group receiving 0.05% atropine nightly had a significantly lower 2‐year cumulative incidence of myopia (28.4% vs. 53.0%) and a reduced myopic shift in SE compared to the placebo group (−0.46 vs. −1.01 D) [12].

The success of public health strategies for myopia prevention depends on premyopic children's adherence to follow‐up ophthalmic care. However, the proportion of preschool children who regularly visit ophthalmologists for comprehensive eye care, including cycloplegic refraction, remains largely unknown. Additionally, access to ophthalmic resources remains a concern, particularly in rural areas.

To address this gap and evaluate the impact of premyopia referral, we conducted a population‐based follow‐up study in Yilan, Taiwan, in 2023, involving two cohorts of schoolchildren who had participated in the YMVIP, a preschool screening programme, before and after the introduction of premyopia referral in 2022. In this study, we analysed the data from schoolchildren with premyopia at baseline, compared annual changes in SE refractive error between these two cohorts, and assessed the extent of adherence to follow‐up ophthalmic care amongst premyopic preschoolers, along with the factors influencing their adherence.

## 2 Methods

### 2.1 Study Design and Participants

To obtain longitudinal data on refractive changes before and after elementary school entry, the YMVIP preschool screening results from the 2021 and 2022 school years were linked to data from a countywide, school‐based study conducted between December 2023 and March 2024 amongst primary‐grade students (first and second graders) in 15 randomly selected elementary schools in Yilan County. In 2022, a premyopia referral policy was officially implemented as part of YMVIP. Accordingly, the first‐grade cohort—preschoolers at the time of policy implementation—is referred to as the intervention cohort, while the second‐grade cohort—who completed preschool screening prior to the policy's initiation—is referred to as the comparison cohort. Both the baseline (preschool) and follow‐up (elementary school) screening programmes included eye examinations and questionnaire surveys. The methodology for preschool screening in the YMVIP has been described previously [11]. In brief, all 5–6‐year‐old kindergarteners in their final year of preschool education in Yilan County were invited to participate in the myopia screening programme during the fall semester (August to December). Participants identified during the screening with reduced VA, myopia, strabismus or other ocular abnormalities were referred for further ophthalmic care. Starting from the 2022 school year, the screening programme expanded its referral criteria to include premyopia. Since then, premyopic preschoolers without other ocular abnormalities have been referred to ophthalmologists for monitoring of refractive error progression. For the follow‐up study, 15 out of 78 elementary schools were randomly selected using probability‐proportional‐to‐size sampling from all 12 townships in Yilan County, including 2 suburban and 10 rural townships. Written informed consent was obtained from both the primary‐grade schoolchildren and their parents. The study was approved by the Institutional Review Board of National Yang Ming Chiao Tung University Hospital (approval identifier: 2022A024) and was conducted in accordance with the tenets of the Declaration of Helsinki.

### 2.2 Eye Examination

All eye examination procedures in both the preschool and elementary school screening programmes adhered to the standardised operating procedures established by the YMVIP organising committee [11]. Briefly, on‐campus eye examinations, including assessments of the external eye, ocular alignment and fundus, were conducted by qualified ophthalmologists. VA was tested by school nursing staff, and refractive status was assessed using an autorefractor after cycloplegia was achieved. Cycloplegia was induced by administering three drops of a combination of tropicamide 0.5% and phenylephrine hydrochloride 0.5%, with a 5‐min interval between each drop. Approximately 30 min after the first drop, the pupillary light reflex was assessed. Cycloplegia was considered complete when the pupil no longer responded to penlight stimulation. If the pupil continued to constrict, an additional cycloplegic drop was administered, followed by a 10‐min wait before conducting cycloplegic autorefraction using an autorefractor (Topcon KR‐1, Tokyo, Japan). Refractive error was calculated by averaging three consecutive autorefraction measurements for each participant. The SE was calculated by adding the spherical power to half of the cylindrical power. Myopia was defined as an SE refractive error of −0.5 D or more myopic in either eye. Those who were not myopic at baseline but became myopic at follow‐up were classified as having incident myopia. In this study, premyopia was defined as an SE refractive error > −0.5 D and ≤ +0.75 D in the eye with the lower SE refractive error for each participant.

### 2.3 Questionnaire Survey

In both the preschool and elementary school screenings, a questionnaire was completed by one parent or the primary caregiver of each participant to collect information regarding caregiver details, the child's medical history, treatments for ocular conditions, time spent on homework (reading, writing, drawing or playing musical instruments), screen time (television, smartphones, computers, tablets or video games) and participation in after‐school outdoor activities on both weekdays and weekends [11]. Additionally, the elementary school questionnaire gathered information about attendance at after‐school tutorial programmes. These questions were closed‐ended and had responses of yes/no options or a list of ordered choices. Respondents selected the most appropriate answer for each item. Children were considered to exhibit after‐school myopiogenic behaviour or lifestyle if their caregivers reported that they spent ≥ 1 h/day on screen‐based devices on weekdays or ≥ 2 h/day on weekends, or engaged in outdoor activities for < 30 min/day on weekdays or < 2 h/day on weekends.

### 2.4 Data Analysis

Only schoolchildren who were identified as premyopic during their preschool examination were recruited for data analysis in this study. For first graders (intervention cohort), data from their elementary school examinations were linked to their preschool data from 2022 based on ID number, while for second graders (comparison cohort), data were linked to their preschool data from 2021. Refractive data from the eye with the lower SE refractive error (the study eye) at the preschool examination were used for the analysis of SE refractive error changes throughout the follow‐up period. The follow‐up period was defined as the interval between the baseline examination at preschool and the follow‐up examination at elementary school. The annual progression rate of SE refractive error was calculated by dividing the change in SE by the exact number of days in the follow‐up period, then multiplying the result by 365. The incidence density or rate of myopia was determined as the number of incident cases divided by the total follow‐up time for all children at risk within the study cohort. Premyopia referral policy for ophthalmic care was officially implemented since the 2022 school year. Adherence to follow‐up eye care was defined as having visited an ophthalmologist at least once within the past year amongst those who received an ophthalmic referral during preschool screening.

Based on data from questionnaire surveys conducted at preschool and elementary school, eligible participants were classified into subgroups according to the change patterns in each myopia‐related lifestyle or behaviour item: ‘maintenance of myopiogenic behaviors’, ‘improvement’, ‘maintenance of protective behaviors’ and ‘deterioration’. For example, regarding time spent on afterschool outdoor activities on weekends, if a child was reported to spend < 2 h/day in both the preschool and elementary school surveys, he or she was classified into the subgroup ‘maintenance of myopiogenic behaviors’. Conversely, if a child was reported to spend ≥ 2 h/day outdoors on weekends in both preschool and elementary school surveys, he or she was classified into the subgroup ‘maintenance of protective behaviors’. A child was classified as showing ‘improvement’ if he or she was reported to spend < 2 h/day outdoors at preschool but ≥ 2 h/day outdoors at elementary school. On the other hand, a child was classified as showing ‘deterioration’ if they were reported to spend more time outdoors on weekends in the past than at present.

Continuous variables were expressed as mean ± standard deviation (SD), while categorical variables were presented as percentages. Differences in continuous data between the two groups were assessed using Student's *t* test; analysis of variance and Pearson's chi‐square test were used for comparing categorical data. Associated factors for the annual SE progression rate and nonadherence to follow‐up visits were analysed using linear and logistic regression models, respectively. Variables with a *p* value of less than 0.1 in the univariable analyses were included in the multivariable logistic or linear regression model using a stepwise approach. Odds ratios (ORs) and their 95% confidence intervals (CIs) were calculated. Statistical significance was inferred at a two‐sided *p* < 0.05. All statistical analyses were conducted using IBM SPSS Statistics version 26 (IBM Corp., Armonk, NY, USA).

## 3 Results

**FIGURE 1:**
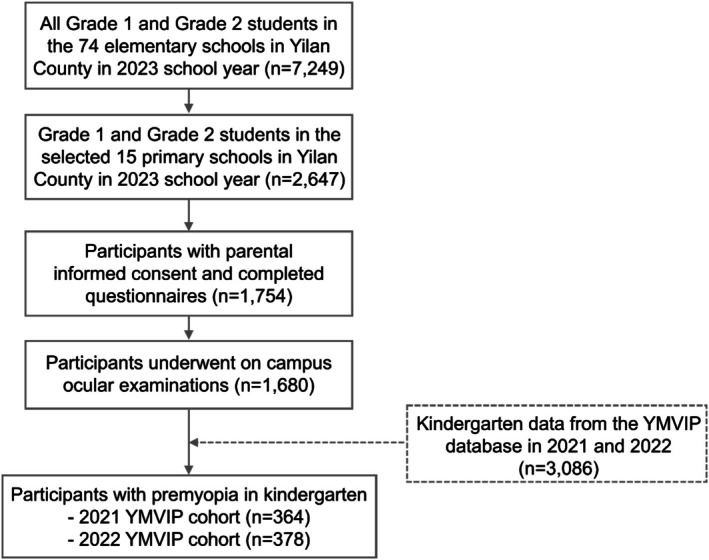
Flowchart showing the recruitment process for this study. YMVIP, Yilan Myopia Prevention and Vision Improvement Program.

From December 2023 to March 2024, a total of 1680 primary‐grade schoolchildren provided informed consent, completed questionnaires and underwent eye examinations. Amongst these, data from 1554 children were successfully linked to their preschool surveys, and 742 of these children were identified as having premyopia at baseline. Figure 1 illustrates the flowchart of participant eligibility and inclusion in the study. When compared to other premyopic preschoolers in the 2021 and 2022 YMVIP cohorts who were not selected for the elementary school follow‐up study (*n* = 2344), these 742 eligible participants were more likely to be boys (57.8% vs. 53.4%, *p* = 0.039), come from the 2022 YMVIP cohort (50.9% vs. 46.4%, *p* = 0.030) and have mild hyperopia (54.3% vs. 50.0%, p = 0.039). The mean SE refractive error of the eligible participants was 0.39 ± 0.32 D, which was significantly more hyperopic than that of the nonparticipants (0.36 ± 0.33 D, *p* = 0.036).

Amongst the 742 primary‐grade participants identified as premyopic during preschool, the annual progression rate of SE refractive error was −0.19 ± 0.39 D/year. A total of 194 (26.14%) incident cases of myopia were observed, with an incidence density of 14.80 per 100 person‐years (95% CI: 12.88–16.72). Table 1 provides a comparison of examination results and questionnaire data between the intervention cohort (*n* = 378) and comparison cohort (*n* = 364). The baseline ages were comparable between the two cohorts (5.65 ± 0.30 vs. 5.67 ± 0.30 years, *p* = 0.481). However, the follow‐up intervals differed significantly (463.45 ± 36.60 vs. 833.17 ± 28.60 days, *p* < 0.001), resulting in a corresponding difference in age at follow‐up (6.92 ± 0.30 vs. 7.94 ± 0.29 years, *p* < 0.001). No significant differences in refractive characteristics at baseline were observed. However, compared to the intervention cohort, participants in the comparison cohort were more likely to attend tutorial programmes for ≥ 1 semester (10.6% vs. 93.2%, *p* < 0.001), attend tutorials ≤ 3 days/week (5.6% vs. 13.4%, *p* < 0.001) and receive cycloplegic eyedrop treatment (15.6% vs. 23.1%, *p* = 0.027). In contrast, participants in the intervention cohort were more likely to receive an ophthalmic referral (82.5% vs. 17.9%, *p* < 0.001) and seek ophthalmic care in the past year (63.2% vs. 53.8%, *p* = 0.006). Regarding mean SE at baseline, children who received an ophthalmic referral were significantly less hyperopic than those who did not (0.32 ± 0.34 vs. 0.47 ± 0.28 D, *p* < 0.001). Similarly, those who received ophthalmic care in the past year were also significantly less hyperopic compared to those who did not (0.35 ± 0.34 vs. 0.45 ± 0.27 D, *p* < 0.001). Furthermore, those who received cycloplegic eyedrop treatment for ocular growth control—without stratification by agent concentration, formulation, or duration of action due to data limitations—exhibited significantly lower hyperopia than those who did not (0.26 ± 0.37 vs. 0.42 ± 0.30 D, *p* < 0.001). Amongst 312 first graders who received an ophthalmic referral during preschool, 107 (34.3%) did not adhere to follow‐up eye care in the past year prior to elementary school screening.

**TABLE 1:** Comparisons between two cohorts with baseline premyopia.

	All primary‐grade children with baseline premyopia (*n* = 742)	Intervention cohort (*n* = 378)	Comparison cohort (*n* = 364)	*p*
Age at baseline age, year	5.65 (0.30)	5.65 (0.30)	5.67 (0.30)	0.481
Age at follow‐up, year	7.42 (0.59)	6.92 (0.30)	7.94 (0.29)	< 0.001
Follow‐up interval, day	644.82 (187.86)	463.45 (36.60)	833.17 (28.60)	< 0.001
Gender, *n* (%)				
Girl	313/742 (42.2%)	158/378 (41.8%)	155/364 (42.6%)	0.829
Boy	429/742 (57.8%)	220/378 (58.2%)	209/364 (57.4%)	
Location of school, *n* (%)				
Rural area	316/742 (42.6%)	173/378 (45.8%)	143/364 (39.3%)	0.074
Suburban area	426/742 (57.4%)	205/378 (54.2%)	221/364 (60.7%)	
Number of myopic parents, *n* (%)				0.719
0	78/718 (10.9%)	37/370 (10.0%)	41/348 (11.8%)	
1	262/718 (36.5%)	138/370 (37.3%)	124/348 (35.6%)	
2	378/718 (52.6%)	195/370 (52.7%)	183/348 (52.6%)	
Education level of parents, *n* (%)				0.488
High school or less	224/739 (30.3%)	118/375 (31.5%)	106/364 (29.1%)	
College or above	515/739 (69.7%)	257/375 (68.5%)	258/364 (70.9%)	
Baseline lifestyle				
Screen time on weekdays (h/day)				0.896
< 1	381/738 (51.6%)	195/376 (51.9%)	186/362 (51.4%)	
≥ 1	357/738 (48.4%)	181/376 (48.1%)	176/362 (48.6%)	
Screen time on weekends (h/day)				0.649
< 2	332/739 (44.9%)	172/376 (45.7%)	160/363 (44.1%)	
≥ 2	407/739 (55.1%)	204/376 (54.3%)	203/363 (55.9%)	
Outdoor time on weekdays (min/day)				0.101
< 30	497/738 (67.3%)	263/375 (70.1%)	234/363 (64.5%)	
≥ 30	241/738 (32.7%)	112/375 (29.9%)	129/363 (35.5%)	
Outdoor time on weekends (h/day)				0.231
< 2	486/739 (65.8%)	255/376 (67.8%)	231/363 (63.6%)	
≥ 2	253/739 (32.2%)	121/376 (32.2%)	132/363 (36.4%)	
*Lifestyle/behaviour change before and after school entry*				
Screen time change on weekdays (myopiogenic: ≥ 1 h/day)				0.240
Maintain myopiogenic behaviour	246/732 (33.6%)	131/372 (35.2%)	115/360 (31.9%)	
Deterioration	108/732 (14.8%)	48/372 (12.9%)	60/360 (16.7%)	
Improvement	146/732 (19.9%)	81/372 (21.8%)	65/360 (18.1%)	
Maintain protective	232/732 (31.7%)	112/372 (30.1%)	120/360 (33.3%)	
Screen time change on weekends (myopiogenic: ≥ 2 h/day)				0.472
Maintain myopiogenic behaviour	266/732 (36.3%)	141/372 (37.9%)	125/360 (34.7%)	
Deterioration	136/732 (18.6%)	61/372 (16.4%)	75/360 (20.8%)	
Improvement	92/732 (12.6%)	47/372 (12.6%)	45/360 (12.5%)	
Maintain protective	238/732 (32.5%)	123/372 (33.1%)	115/360 (31.9%)	
Outdoor time change on weekdays (myopiogenic: < 30 min/day)				0.276
Maintain myopiogenic behaviour	354/730 (48.5%)	182/370 (49.2%)	172/360 (47.8%)	
Deterioration	138/730 (18.9%)	78/370 (21.1%)	60/360 (16.7%)	
Improvement	92/730 (12.6%)	43/370 (11.6%)	49/360 (13.6%)	
Maintain protective	146/730 (20.0%)	67/370 (18.1%)	79/360 (21.9%)	
Outdoor time change on weekends (myopiogenic: < 2 h/day)				0.098
Maintain myopiogenic behaviour	369/732 (50.4%)	187/371 (50.4%)	182/361 (50.4%)	
Deterioration	113/732 (15.4%)	66/371 (17.8%)	47/361 (13.0%)	
Improvement	121/732 (16.5%)	51/371 (13.7%)	70/361 (19.4%)	
Maintain protective	129/732 (17.6%)	67/371 (18.1%)	62/361 (17.2%)	
*Tutoring programme attendance (after‐school and weekend)*				
Tutorial program type				0.445
In‐campus	151/654 (23.1%)	73/334 (21.9%)	78/320 (24.4%)	
Off‐campus	503/654 (76.9%)	261/334 (78.1%)	242/320 (75.6%)	
Duration of attending tutoring program (semester)				**< 0.001**
< 1	318/653 (48.7%)	296/331 (89.4%)	22/322 (6.8%)	
≥ 1	335/653 (51.3%)	35/331 (10.6%)	300/322 (93.2%)	
Weekly hours of attending tutoring program (h/week)				
< 10	198/658 (30.1%)	91/336 (27.1%)	107/322 (33.2%)	0.086
≥ 10	460/658 (69.9%)	245/336 (72.9%)	215/322 (66.8%)	
Attendance on weekdays (days/week)				**< 0.001**
≤ 3 (including no attendance)	63/670 (9.4%)	19/342 (5.6%)	44/328 (13.4%)	
≥ 4	607/670 (90.6%)	323/342 (94.4%)	284/328 (86.6%)	
Attendance on weekends				0.660
No weekend attendance	574/601 (95.5%)	294/309 (95.1%)	280/292 (95.9%)	
≥ 1 days/weekend	27/601 (4.5%)	15/309 (4.9%)	12/292 (4.1%)	
*Baseline refractive error and results of ophthalmic referral*				
Baseline SE, mean (SD)	0.39 (0.32)	0.38 (0.33)	0.40 (0.31)	0.214
Baseline SE distribution, *n* (%)				0.434
Emmetropia	339/742 (45.7%)	178/378 (47.1%)	161/364 (44.2%)	
Mild hyperopia	403/742 (54.3%)	200/378 (52.9%)	203/364 (55.8%)	
Ophthalmic referral at baseline, *n* (%)				**< 0.001**
Yes	377/742 (50.8%)	312/378 (82.5%)	65/364 (17.9%)	
No	349/742 (47.0%)	53/378 (14.0%)	296/364 (81.3%)	
Unknown	16/742 (2.2%)	13/378 (3.4%)	3/364 (0.8%)	
Seeking ophthalmic care over the past year, *n* (%)				**0.006**
Yes	435/742 (58.6%)	239/378 (63.2%)	196/364 (53.8%)	
No	296/742 (39.9%)	131/378 (34.7%)	165/364 (45.3%)	
Unknown	11/742 (1.5%)	8/378 (2.1%)	3/364 (0.8%)	
Cycloplegic treatment over the past year, *n* (%)				**0.027**
Yes	143/742 (19.3%)	59/378 (15.6%)	84/364 (23.1%)	
No	583/742 (78.6%)	312/378 (82.5%)	271/364 (74.5%)	
Unknown	16/742 (2.2%)	7/378 (1.9%)	9/364 (2.5%)	

*Note: p* < 0.05 are in bold type.

Abbreviations: SD = standard deviation, SE = spherical equivalent.

Table 2 presents the refractive outcomes between these two cohorts with preschool premyopia. The annual progression rate of SE refractive error was significantly slower in the intervention cohort (−0.15 ± 0.46 vs. −0.22 ± 0.29 D/year, *p* = 0.009). The comparison cohort demonstrated a higher rate of incident myopia (34.3% vs. 18.3%, *p* < 0.001) and showed marginally higher incidence density (15.04 per 100 person‐years, 95% CI: 12.61–17.47) than the intervention cohort (14.38 per 100 person‐years, 95% CI: 11.24–17.52).

**TABLE 2:** Refractive outcomes between two cohorts of premyopic preschoolers.

	All schoolchildren with baseline premyopia (*n* = 742)	Intervention cohort (*n* = 378)	Comparison cohort (*n* = 364)	*p*
SE progression rate (D/year), mean (SD)	−0.19 (0.39)	−0.15 (0.46)	−0.22 (0.29)	**0.009**
Incident myopia, *n* (%)	194/742 (26.14%)	69/378 (18.25%)	125/364 (34.34)	**< 0.001**
Incidence density/(100‐person‐year)	14.80 (95% CI: 12.88–16.72)	14.38 (95% CI: 11.24–17.52)	15.04 (95% CI: 12.61–17.47)	n.s.

*Note: p* < 0.05 are in bold type.

Abbreviations: D = diopter, SD = standard deviation, SE = spherical equivalent.

Table 3 summarises the results of stepwise multiple linear regression analyses for myopic shift in SE refractive error. Significant associated factors included having a myopic mother (*β* = −0.116, *p* = 0.004), having a myopic father (*β* = −0.074, *p* = 0.027), not receiving an ophthalmic referral at baseline (*β* = 0.070, *p* = 0.030), seeking ophthalmic care in the past year (*β* = −0.119, *p* < 0.001), spending < 30 min on after‐school outdoor activities during weekdays at baseline (*β* = 0.085, *p* = 0.009) and attending weekend tutorial programmes (*β* = −0.188, *p* = 0.017). The annual progression rates of SE refractive error were −0.01 ± 0.41, −0.16 ± 0.36, −0.25 ± 0.39 and −0.31 ± 0.45 D/year in premyopic children with no myopic parents, one myopic parent, two myopic parents and two highly myopic parents, respectively (*p* < 0.001).

**TABLE 3:** Association factors for myopic shift amongst schoolchildren with baseline premyopia (*n* = 742).

	Multivariate (stepwise)					
	*β*	Standard error	95% CI	*p*	Tolerance	VIF
Mother with myopia	−0.116	0.040	−0.194 to −0.038	**0.004**	0.978	1.023
Father with myopia	−0.074	0.033	−0.139 to −0.008	**0.027**	0.990	1.010
Ophthalmic referral	0.070	0.032	0.007 to 0.133	**0.030**	0.951	1.052
Ophthalmology visit	−0.119	0.033	−0.183 to −0.055	**< 0.001**	0.946	1.057
*Baseline lifestyle*						
Outdoor time on weekdays (min/day)						
< 30	Ref					
≥ 30	0.085	0.033	0.021 to 0.149	**0.009**	0.990	1.010
*Tutoring program (after‐school and weekend)*						
Days of tutoring on weekends						
0 day/week	Ref					
1 or 2 days/week	−0.188	0.078	−0.341 to −0.034	**0.017**	0.989	1.011

*Note: p* < 0.05 are in bold type.

Abbreviations: CI = confident interval, VIF = variance inflation factor.

Table 4 presents the results of simple and stepwise multiple logistic regression analyses on nonadherence to follow‐up eye care amongst premyopic preschoolers identified in the intervention cohort. In summary, nonadherence was associated with baseline SE refractive error (adjusted OR = 3.77, 95% CI: 1.69–8.41) and attending kindergartens in rural areas (adjusted OR = 2.01, 95% CI: 1.23–3.30).

**TABLE 4:** Factors associated with nonadherence to follow‐up eye care amongst premyopic preschoolers who received an ophthalmic referral in 2022 (*n* = 312).

	Simple logistic regression			Stepwise multiple logistic regression		
	Odds ratio	95% CI	*p*	Odds ratio	95% CI	*p*
Baseline spherical equivalent (D)	3.25	1.49–7.05	**0.003**	3.77	1.69–8.41	**0.001**
Gender						
Girl	Ref					
Boy	0.67	0.42–1.08	0.098		—	
Location of kindergarten						
Suburban area	Ref			Ref		
Rural area	1.76	1.10–2.84	**0.019**	2.01	1.23–3.30	**0.005**
Kindergarten type						
Public	Ref					
Private	0.82	0.51–1.31	0.410			
Parental factors						
Mother with myopia	0.83	0.48–1.45	0.516			
Father with myopia	0.95	0.58–1.55	0.833			
Number of myopic parents						
0	Ref				—	—
1	0.47	0.20–1.08	0.076		—	—
2	0.57	0.25–1.28	0.170		—	—
Number of highly myopic parents						
0	Ref					
1	0.55	0.32–0.97	**0.038**			
2	0.77	0.14–4.27	0.760			
Education level of parents						
High school or less	Ref					
College or above	0.85	0.51–1.41	0.525			
*Baseline lifestyle*						
Screen time on weekdays (h/day)						
< 1	Ref					
≥ 1	0.96	0.60–1.55	0.881			
Screen time on weekends (h/day)						
< 2	Ref					
≥ 2	1.04	0.65–1.67	0.871			
Outdoor time on weekdays (min/day)						
< 30	Ref					
≥ 30	0.73	0.45–1.19	0.204			
Outdoor time on weekends (h/day)						
< 2	Ref					
≥ 2	0.81	0.50–1.33	0.407			

*Note: p* < 0.05 are in bold type.

Abbreviations: D = diopter, CI = confident interval.

## 4 Discussion

This study provides population‐based evidence on the impact of early referral for premyopia on myopia development after school entry. Following the inclusion of premyopia as a referral criterion in the 2022 preschool screening, the myopic shift in SE refractive error slowed by 31.8%; although the incidence density of myopia did not decrease significantly. Receiving an ophthalmic referral during preschool is a protective factor against myopic SE shift in premyopic children. More than 60% of children in the intervention cohort that received an ophthalmic referral during preschool adhered to follow‐up eye care within the past year. Nonadherence was associated with a more hyperopic SE refractive error at baseline and residence in rural areas. This longitudinal study may serve as a useful reference to enhance premyopia management before school entry.

Premyopia, particularly during the preschool years, represents a critical window for interventions aimed at preventing high myopia. A prospective cohort study reported that 53.9% of children who developed myopia at an early school age (7‐ or 8‐year‐olds) eventually progressed to high myopia in adulthood [1]. Both environmental and pharmaceutical interventions have been shown to be effective in slowing the myopic shift in SE refractive error amongst premyopic children [5, 12]. A subgroup analysis of the Recess Outside Classroom (ROC) study—a prospective, comparative, interventional study employing an educational policy to increase outdoor time at school—revealed that premyopic schoolchildren in the ROC group had a significantly slower myopic shift than the controls (50% slower: −0.20 ± 0.60 vs. −0.40 ± 0.66 D/year, *p* = 0.017) [5]. Additionally, the 12‐month results of the LAMP2 study showed that the premyopic participants experienced myopic shifts of −0.11 D (95% CI, −0.18 to −0.04) in the 0.05% atropine group, −0.38 D (95% CI, −0.46 to −0.30) in the 0.01% atropine group and −0.55 D (95% CI, −0.64 to −0.45) in the placebo group (*p* < 0.001) [12]. The 12‐month reduction in the myopic shift was 80% when comparing the 0.05% atropine group to the placebo group, and 71% when comparing the 0.05% and 0.01% atropine groups [12]. These positive findings highlight the importance of early implementation of these preventive interventions for premyopic children to delay myopia development.

In our study, both cohorts of premyopic children participated in eye care programmes that promoted outdoor activities from kindergarten (YMVIP) through elementary school (Tian‐Tian 120 outdoor programme). The overall myopic shift amongst all 742 eligible participants was −0.19 ± 0.39 D/year, comparable to that observed in the abovementioned ROC group (−0.20 ± 0.60 D/year). Notably, despite the presence of these existing eye care programmes (YMVIP and Tian‐Tian 120 outdoor programme), the myopic shift further slowed around elementary school entry following the implementation of a new referral policy for premyopic preschoolers in 2022. The intervention cohort exhibited a significantly lower myopic shift compared to the comparison cohort, with a 31.8% reduction (−0.15 ± 0.46 vs. −0.22 ± 0.29 D/year, *p* = 0.009). Multiple regression analysis shows that receiving an ophthalmic referral at baseline was significantly associated with slower myopic SE shift (*β* = 0.070, *p* = 0.030). Since the 2022 school year, YMVIP has asked parents or caregivers who received a referral to take their premyopic children for ophthalmic care and refractive error monitoring. This process helps inform parents or caregivers about their children's risk of myopia development and allows them to receive relevant healthcare guidance at ophthalmic clinics. However, the 31.8% reduction in myopic shift observed in our study was smaller than that reported in previous interventional studies (50% in the ROC study and 71%–80% in the LAMP2 study) [5, 12], which may partially explain the lack of a significant difference in myopia incidence density between the two cohorts. Even so, promoting awareness of premyopia and its associated risk of myopia through ophthalmic referrals may further enhance current myopia prevention strategies in kindergartens and elementary schools.

In addition to parental awareness of myopia risk, certain parental factors may contribute to early‐onset myopia, especially the presence of parental myopia, a well‐documented risk factor in published literature [13]. A pooled analysis of 3 population‐based studies from the United States, Singapore and Australia confirmed a significant association between parental myopia and preschool myopia with ORs of 1.42 (95% CI, 1.20–1.68), 2.70 (95% CI, 2.19–3.33) and 3.39 (95% CI, 1.99–5.78) for children with one myopic parent, two myopic parents and two juvenile‐onset myopic parents, respectively, compared with children without parental myopia [14]. Our study also found that a greater number of myopic parents was associated with a more rapid annual myopic shift in SE refractive error. Therefore, premyopic preschoolers with one or both myopic parents may require more proactive and effective interventions to prevent or delay myopia onset.

Myopia prevention strategies in Taiwan primarily focus on environmental and behavioural interventions. Children are advised to undergo ophthalmic exams once or twice a year, limit screen time to ≤ 1 h per day, spend at least 2 h outdoors daily and take a 10‐min break every 30 min of near work [10]. Despite promising findings from the LAMP2 study [12], there remains no consensus on pharmacological interventions for premyopic children. In Taiwan, compared to policy‐driven eye care programmes promoting outdoor activities, topical cycloplegic agents, such as low‐dose atropine eyedrops, are not widely prescribed to non‐myopic children for ocular growth control and myopia prevention. In our study, amongst 435 children who were premyopic at baseline and had received at least one ophthalmic exam in the past year, only 143 (32.9%) reported using cycloplegic eyedrops. Those who received cycloplegic treatment had baseline SE values closer to myopia compared to those who did not (0.26 ± 0.37 D vs. 0.42 ± 0.30 D, *p* < 0.001). Notably, more than 50% of all eligible participants in this study had two parents with myopia. If all the premyopic preschoolers with additional risk factors, such as having two myopic parents, receive a combination of both environmental and pharmacological interventions, the risk of myopia development may be further minimised [5, 12].

Comprehensive eye examinations play a crucial role in monitoring SE progression, especially in young children at high risk of developing myopia. In our preschool screening, 9.2% of children were already myopic and 46.1% were classified as premyopic. In 2022, most premyopic preschoolers in the YMVIP screening programme were referred for ophthalmic care. Caregivers of these children received a referral sheet detailing the child's refractive measurements, the definition of premyopia and a recommendation for follow‐up with an ophthalmologist within three months. However, we observed that only 63.2% of children in the intervention cohort with an initial ophthalmic referral adhered to follow‐up visits over the past year. In addition, nonadherence was more prevalent amongst children with a more hyperopic SE at baseline. Previous studies have shown that a sufficient hyperopic reserve at baseline decreases the likelihood of developing myopia [15]. Conversely, children with lower hyperopic reserves have a greater risk of developing myopia and are more likely to receive regular follow‐up eye care. This tendency may partially explain the observed link between myopic SE shift and adherence to ophthalmic care in our study. Apart from baseline SE, nonadherence was also significantly associated with urbanisation level of residence. It is important to incorporate cycloplegic autorefraction into school‐based screening programmes, as this can help monitor refractive status, particularly amongst children in rural areas.

Attending after‐school tutorial programmes is common amongst Taiwanese schoolchildren and has been reported as a risk factor of myopia [16, 17]. Based on our results, rapid myopic SE shift was also associated with attending tutorial programmes on weekends after school entry. This observed association may have resulted from the appearance of academic pressure, increased near work and reduced outdoor time. Consistent with previous studies, spending enough time outdoors was identified as a protective factor against myopic SE shift amongst non‐myopic preschoolers in our study [7, 18]. However, after the onset of myopia, the effect of increased outdoor time on slowing myopia progression was not significant or was smaller than that of atropine treatment amongst myopic children [18, 19, 20]. These findings highlight the importance of early promotion of outdoor activities in kindergartens, when most children have not yet developed myopia.

There are some limitations in the current study. First, we adopted probability‐proportional‐to‐size sampling to ensure a representative sample of primary‐grade schoolchildren in the follow‐up study. However, amongst premyopic preschoolers at baseline, the distribution of baseline refractive status was not well balanced between study participants and those who were not selected for follow‐up. The former had a more hyperopic mean SE in preschool than the latter (0.39 ± 0.32 vs. 0.36 ± 0.33 D, *p* = 0.036). Although it may not be clinically significant—given that the minimum measurement increment of the autorefractor is 0.25 D—the myopic shift and incidence amongst premyopic preschoolers could have been underestimated in this study. Second, the follow‐up intervals differed between the two cohorts in our study, resulting in a higher age at follow‐up in the comparison cohort. This discrepancy may have led to an overestimation of the effectiveness of premyopic referral. To address this potential bias, we standardised the outcome measurements using annualised metrics—specifically, myopia incidence expressed per 100 person‐years and annual myopic shift expressed in diopters per year. Furthermore, our multivariate analyses with age adjustment consistently demonstrated that ophthalmic referral at baseline was significantly associated with a reduced annual myopic shift. These findings support the robustness of our conclusion, despite differences in follow‐up duration. Third, information about lifestyle, medical history and healthcare‐seeking behaviour was gathered through a caregiver‐administered questionnaire in this study. However, specific details about the concentrations and formulations of cycloplegic eyedrops used for ocular growth control were not available. Although most questions focused on daily routines and frequent events, minimising the likelihood of recall bias, self‐report bias remains inevitable. To enhance accuracy, further studies should incorporate wearable devices for objective measurement of near work and outdoor time and link data to clinical charts for treatment regimen records.

In conclusion, early identification and ophthalmic referral may further slow the myopic SE shift amongst premyopic preschoolers participating in an eyecare programme that promotes outdoor time. Additionally, parental myopia and attending a weekend tutorial programme after entering elementary school are associated with a faster myopic SE shift in premyopic preschoolers. Children with greater hyperopic reserve at baseline and those living in rural townships were less likely to adhere to follow‐up ophthalmic care. Our findings highlight the need to pay closer attention to premyopic children, especially those with myopic parents and heavy academic burdens.

## Disclosure

During the preparation of this work, the authors used ChatGPT (OpenAI) to improve language and readability. After using this tool, the authors reviewed and edited the content as needed and take full responsibility for the final version of the manuscript.

## Conflicts of Interest

The authors declare no conflicts of interest.

## Supporting information


**Table S1:** Comparisons between premyopic preschoolers who participated in or were not selected for follow‐up study.

**Table S2:** Survey questions and responses in the baseline questionnaire.

**Table S3:** Survey questions and responses in the follow‐up questionnaire.

## Data Availability

The data that support the findings of this study are available from the corresponding author upon reasonable request.

## References

[1] Y. Hu , X. Ding , X. Guo , Y. Chen , J. Zhang , and M. He , “Association of Age at Myopia Onset With Risk of High Myopia in Adulthood in a 12‐Year Follow‐up of a Chinese Cohort,” JAMA Ophthalmology 138 (2020): 1129–1134. 32940622 10.1001/jamaophthalmol.2020.3451 PMC7499247

[2] K. S. Naidoo , T. R. Fricke , K. D. Frick , et al., “Potential Lost Productivity Resulting from the Global Burden of Myopia: Systematic Review, Meta‐analysis, and Modeling,” Ophthalmology 126 (2019): 338–346. 30342076 10.1016/j.ophtha.2018.10.029

[3] J. S. Wolffsohn , D. I. Flitcroft , K. L. Gifford , et al., “IMI ‐ Myopia Control Reports Overview and Introduction,” Investigative Ophthalmology & Visual Science 60, no. 3 (2019): M1–M19. 30817825 10.1167/iovs.18-25980 PMC6735780

[4] D. I. Flitcroft , M. He , J. B. Jonas , et al., “IMI ‐ Defining and Classifying Myopia: A Proposed Set of Standards for Clinical and Epidemiologic Studies,” Investigative Ophthalmology & Visual Science 60 (2019): M20–M30. 30817826 10.1167/iovs.18-25957 PMC6735818

[5] P. C. Wu , C. L. Tsai , and Y. H. Yang , “Outdoor Activity During Class Recess Prevents Myopia Onset and Shift in Premyopic Children: Subgroup Analysis in the Recess Outside Classroom Study,” Asia‐Pacific Journal of Ophthalmology 14 (2025): 100140. 39805427 10.1016/j.apjo.2025.100140

[6] C. Y. Wang , N. W. Hsu , Y. C. Yang , Y. L. Chen , M. P. Shyong , and D. C. Tsai , “Premyopia at Preschool Age: Population‐based Evidence of Prevalence and Risk Factors From a Serial Survey in Taiwan,” Ophthalmology 129 (2022): 880–889. 35331752 10.1016/j.ophtha.2022.03.017

[7] P. C. Wu , C. L. Tsai , H. L. Wu , Y. H. Yang , and H. K. Kuo , “Outdoor Activity During Class Recess Reduces Myopia Onset and Progression in School Children,” Ophthalmology 120 (2013): 1080–1085. 23462271 10.1016/j.ophtha.2012.11.009

[8] M. He , F. Xiang , Y. Zeng , et al., “Effect of Time Spent Outdoors at School on the Development of Myopia Among Children in China: A Randomized Clinical Trial,” JAMA 314 (2015): 1142–1148. 26372583 10.1001/jama.2015.10803

[9] P. C. Wu , C. T. Chen , L. C. Chang , et al., “Increased Time Outdoors Is Followed by Reversal of the Long‐Term Trend to Reduced Visual Acuity in Taiwan Primary School Students,” Ophthalmology 127 (2020): 1462–1469. 32197911 10.1016/j.ophtha.2020.01.054

[10] “Vision Surveillance Survey of Children and Adolescents. The Health Promotion Administration, Ministry of Health and Welfare, Executive Yuan, Republic of China (Taiwan),” 2025, https://www.hpa.gov.tw/Pages/List.aspx?nodeid=45.

[11] Y. C. Yang , N. W. Hsu , C. Y. Wang , M. P. Shyong , and D. C. Tsai , “Prevalence Trend of Myopia after Promoting Eye Care in Preschoolers: A Serial Survey in Taiwan before and during the Coronavirus Disease 2019 Pandemic,” Ophthalmology 129 (2022): 181–190. 34425129 10.1016/j.ophtha.2021.08.013

[12] J. C. Yam , X. J. Zhang , Y. Zhang , et al., “Effect of Low‐Concentration Atropine Eyedrops vs Placebo on Myopia Incidence in Children: The LAMP2 Randomized Clinical Trial,” JAMA 329 (2023): 472–481. 36786791 10.1001/jama.2022.24162 PMC9929700

[13] I. G. Morgan , P. C. Wu , L. A. Ostrin , et al., “IMI Risk Factors for Myopia,” Investigative Ophthalmology & Visual Science 62 (2021): 3. 10.1167/iovs.62.5.3 PMC8083079 33909035

[14] X. Jiang , K. Tarczy‐Hornoch , S. A. Cotter , et al., “Association of Parental Myopia With Higher Risk of Myopia Among Multiethnic Children Before School Age,” JAMA Ophthalmology 138 (2020): 501–509. 32191277 10.1001/jamaophthalmol.2020.0412 PMC7082765

[15] J. Wang , Z. Qi , Y. Feng , et al., “Normative Value of Hyperopia Reserve and Myopic Shift in Chinese Children and Adolescents Aged 3–16 Years,” British Journal of Ophthalmology 108 (2024): 1024–1029. 37709362 10.1136/bjo-2023-323468 PMC11228215

[16] D. C. Tsai , S. Y. Fang , N. Huang , et al., “Myopia Development Among Young Schoolchildren: The Myopia Investigation Study in Taipei,” Investigative Ophthalmology & Visual Science 57 (2016): 6852–6860. 28002845 10.1167/iovs.16-20288

[17] P. W. Ku , A. Steptoe , Y. J. Lai , et al., “The Associations Between Near Visual Activity and Incident Myopia in Children: A Nationwide 4‐Year Follow‐up Study,” Ophthalmology 126 (2019): 214–220. 29934268 10.1016/j.ophtha.2018.05.010

[18] P. C. Wu , C. T. Chen , K. K. Lin , et al., “Myopia Prevention and Outdoor Light Intensity in a School‐Based Cluster Randomized Trial,” Ophthalmology 125 (2018): 1239–1250. 29371008 10.1016/j.ophtha.2017.12.011

[19] L. A. Jones‐Jordan , L. T. Sinnott , S. A. Cotter , et al., “Time Outdoors, Visual Activity, and Myopia Progression in Juvenile‐Onset Myopes,” Investigative Ophthalmology & Visual Science 53 (2012): 7169–7175. 22977132 10.1167/iovs.11-8336 PMC3474591

[20] C. C. Hsu , N. Huang , P. Y. Lin , et al., “Risk Factors for Myopia Progression in Second‐Grade Primary School Children in Taipei: A Population‐Based Cohort Study,” British Journal of Ophthalmology 101 (2017): 1611–1617. 28315834 10.1136/bjophthalmol-2016-309299

