# Supplementary material for: Premyopia Management With Ophthalmic Referral Slows Myopic Shift After School Entry: A Population‐Based Longitudinal Study in Taiwan
Source: Clin Exp Ophthalmol. 2025 Aug 9;53(9):1104–14. doi: 10.1111/ceo.14595 (PMC12747476; doi:10.1111/ceo.14595)
Supplement: Supplementary file 1 — Table S1: Comparisons between premyopic preschoolers who participated in or were not selected for follow‐up study. [file CEO-53-1104-s001.docx]

**Supplementary Table S1.** Comparisons between premyopic preschoolers who participated in or were not selected for follow-up study.

|  | Participants of follow-up study (n=742) | Non-participants (n=2344) | P value |
| --- | --- | --- | --- |
| Gender, n(%) |  |  |  |
| Girls | 313 (42.2%) | 1092 (46.6%) | ***0.039*** |
| Boys | 429 (57.8%) | 1252 (53.4%) |  |
| YMVIP cohort, n (%) |  |  |  |
| 2021 | 364 (49.1%) | 1257 (53.6%) | ***0.030*** |
| 2022 | 378 (50.9%) | 1087 (46.4%) |  |
| Location of kindergartens, n (%) |  |  |  |
| Rural area | 351 (47.3%) | 1190 (50.8%) | 0.098 |
| Suburban area | 391 (52.7%) | 1153 (49.2%) |  |
| Baseline SE, mean (SD) | 0.39 (0.32) | 0.36 (0.33) | ***0.036*** |
| Baseline SE distribution, n (%) |  |  |  |
| Emmetropia | 339 (45.7%) | 1173 (50.0%) | ***0.039*** |
| Mild hyperopia | 403 (54.3%) | 1171 (50.0%) |  |

SD =standard deviation; SE = spherical equivalent.

P values < 0.05 are in bold type.
