# Supplementary material for: Premyopia Management With Ophthalmic Referral Slows Myopic Shift After School Entry: A Population‐Based Longitudinal Study in Taiwan
Source: Clin Exp Ophthalmol. 2025 Aug 9;53(9):1104–14. doi: 10.1111/ceo.14595 (PMC12747476; doi:10.1111/ceo.14595)
Supplement: Supplementary file 2 — Table S2: Survey questions and responses in the baseline questionnaire. [file CEO-53-1104-s003.docx]

**Supplementary Table S2. Survey questions and responses in the baseline questionnaire.**

| **Caregiver’s information** |
| --- |
| - Age, gender, occupation |
| - Do you have myopia? □no, □yes |
| - Education level: □primary school, □junior high school, □senior high school/vocational school, □junior college or university, □graduate school or above |
| - Relationship with the child: □parent, □paternal grandparent, □maternal grandparent, □others______ |
| **Medical history of the child** |
| - Does your child have past ocular history? □no, □yes   - If yes, what is the disease? □congenital glaucoma, □congenital cataract, □retinopathy of premature, □strabismus, □asthma, □ocular trauma, □ocular surgery, □others______ |
| - Did you have your child’s eyes examined by an ophthalmologist over the past year?  □no, □yes |
| - Is your child currently treated for ocular disease? □no, □yes   - If yes, what is the treatment? □cycloplegic agents, □spectacles , □orthokeratology, □patching for amblyopia |
| - When did your child start learning to write? □< 3 years old, □≥ 3 but <4 years old, □≥ 4 but <5 years old, □≥ 5 but <6 years old □≥ 6 years old |
| - In recent one week , how long did your child sleep every night? □<7 hours, □≥ 7 but <8 hours , □≥ 8 but <9 hours, □≥ 9 but <10 hours , □≥10 hours |
| **Lifestyle and near-work habits of the child** |
| - In recent one week, how much time a day did your child spend on doing homework (such as reading, writing, drawing and playing musical instruments)?   - On weekdays: □none, □< 30 minutes, □≥ 30 minutes but < 1 hour, □≥ 1 but < 2 hours, □≥ 2 but< 4 hours, □≥ 4 hours   - On weekends: □none, □< 30 minutes, □≥ 30 minutes but < 1 hour, □≥ 1 but < 2 hours, □≥ 2 but< 4 hours, □≥ 4 hours |
| - In recent one week, how much time a day did your child spend on using screen-based devices (such as watching television and playing smartphones, computers, tablets or video games)?   - On weekdays: □none, □< 30 minutes, □≥ 30 minutes but < 1 hour, □≥ 1 but < 2 hours, □≥ 2 but< 4 hours, □≥ 4 hours   - On weekends: □none, □< 30 minutes, □≥ 30 minutes but < 1 hour, □≥ 1 but < 2 hours, □≥ 2 but< 4 hours, □≥ 4 hours |
| - In recent one week, how much time a day did your child spend on after-school outdoor activities (such as playing balls, swimming or cycling)?   - On weekdays: □none, □< 30 minutes, □≥ 30 minutes but < 1 hour, □≥ 1 but < 2 hours, □≥ 2 but< 4 hours, □≥ 4 hours   - On weekends: □none, □< 30 minutes, □≥ 30 minutes but < 1 hour, □≥ 1 but < 2 hours, □≥ 2 but< 4 hours, □≥ 4 hours |
