# Supplementary material for: Premyopia Management With Ophthalmic Referral Slows Myopic Shift After School Entry: A Population‐Based Longitudinal Study in Taiwan
Source: Clin Exp Ophthalmol. 2025 Aug 9;53(9):1104–14. doi: 10.1111/ceo.14595 (PMC12747476; doi:10.1111/ceo.14595)
Supplement: Supplementary file 3 — Table S3: Survey questions and responses in the follow‐up questionnaire. [file CEO-53-1104-s002.docx]

**Supplementary Table S3. Survey questions and responses in the follow-up questionnaire.**

| **Caregiver’s information** |
| --- |
| - Your relationship with the child:  □mother, □father, □paternal grandparent, □maternal grandparent, □others______ - Age - Gender - Occupation |
| - Education level:   - Child’s father:  □primary school, □junior high school, □senior high school/vocational school, □junior college or university, □graduate school or above   - Child’s mother:  □primary school, □junior high school, □senior high school/vocational school, □junior college or university, □graduate school or above - Do the parents have myopia?  □no_ none has myopia, □yes_ only father has myopia, □yes_ only mother has myopia, □yes_ both have myopia |
| **Medical history of the child** |
| - Does your child have past ocular history?  □no, □yes   - If yes, what is the disease?  □congenital glaucoma, □congenital cataract, □retinopathy of premature, □strabismus, □asthma, □ocular trauma, □ocular surgery, □others______ |
| - Did you have your child’s eyes examined by an ophthalmologist over the past year?  □no, □yes |
| - Has your child received any ophthalmic treatment over the past year?  □no, □yes   - If yes, what kind of the treatment? □long-acting cycloplegic agents, □short-acting cycloplegic agents, □spectacles, □orthokeratology, □patching for amblyopia |
| **Lifestyle, near-work habits and outdoor activity of the child** |
| - In recent one week, how much time a day did your child spend on doing homework (such as reading, writing, drawing and playing musical instruments)?   - On weekdays:  □none, □< 30 minutes, □≥ 30 minutes but < 1 hour, □≥ 1 but < 2 hours, □≥ 2 but< 4 hours, □≥ 4 hours   - On weekends:  □none, □< 30 minutes, □≥ 30 minutes but < 1 hour, □≥ 1 but < 2 hours, □≥ 2 but< 4 hours, □≥ 4 hours - In recent one week, how much time a day did your child spend on using screen-based devices (such as watching television and playing smartphones, computers, tablets or video games)?   - On weekdays:  □none, □< 30 minutes, □≥ 30 minutes but < 1 hour, □≥ 1 but < 2 hours, □≥ 2 but< 4 hours, □≥ 4 hours   - On weekends:  □none, □< 30 minutes, □≥ 30 minutes but < 1 hour, □≥ 1 but < 2 hours, □≥ 2 but< 4 hours, □≥ 4 hours - In recent one week, how much time a day did your child spend on after-school outdoor activities (such as playing balls, swimming or cycling)?   - On weekdays:  □none, □< 30 minutes, □≥ 30 minutes but < 1 hour, □≥ 1 but < 2 hours, □≥ 2 but< 4 hours, □≥ 4 hours   - On weekends:  □none, □< 30 minutes, □≥ 30 minutes but < 1 hour, □≥ 1 but < 2 hours, □≥ 2 but< 4 hours, □≥ 4 hours - In recent one week , how long did your child sleep every night?  □<7 hours, □≥ 7 but <8 hours , □≥ 8 but <9 hours, □≥ 9 but <10 hours , □≥10 hours |
| **Attendance of after-school activity/tutoring** |
| - Does your child attend after-school activity/tutoring programs?  □yes, □no, □not sure   - If yes, what kind of after-school activity? □on-campus care service/club activity, □off-campus care service, □off-campus tutoring program/cram school, □off-campus compound (care and tutoring) program   - If yes, how much time a week does your child spend on after-school activity? □< 5 hours, □≥ 5 hours but < 10 hours, □≥ 10 hours   - If yes, how many days a week does your child attend on these programs on weekdays? □1 day, □2 days, □3 days, □4 days, □5 days, □varies   - If yes, how many days a week does your child attend on these programs on weekends? □1 day, □2 days, □varies   - If yes, how long has your child attended after-school activity? □< 1 semester, □≥ 1 but < 2 semesters, □≥ 2 but < 3 semesters, □≥ 3 semesters |
